# Factors Affecting Mode of Birth in Women With Preexisting Diabetes and Gestational Diabetes: A Retrospective Cohort at a Tertiary Referral Center

**DOI:** 10.1155/2024/5561761

**Published:** 2024-05-31

**Authors:** Theresa Reischer, Sina Prossinger, Anja Catic, Eibhlin Healy, Christian Göbl, Gülen Yerlikaya-Schatten

**Affiliations:** ^1^ Department of Gynaecology and Obstetrics Division of Feto-Maternal Medicine Medical University of Vienna, Vienna, Austria; ^2^ Fetal Medicine Unit Liverpool Women's Hospital, Liverpool, UK

**Keywords:** caesarean section, gestational diabetes mellitus, induction of labor, maternal outcome, mode of birth, pre-existing diabetes

## Abstract

Women with preexisting diabetes and gestational diabetes mellitus (GDM) are at higher risk for adverse maternal and neonatal outcomes. However, there is no consensus on a uniform approach regarding mode of birth (MOB) for all forms of diabetes. The aim of the study is to compare MOB in women with preexisting diabetes and GDM and possible factors influencing it. A retrospective cohort study of women with GDM and preexisting diabetes between 2015 and 2021 at a tertiary referral center was conducted. One thousand three hundred eighty-five singleton pregnancies were included. One thousand twenty-two (74.4%) women had a vaginal birth (VB) and 351 (25.6%) a caesarean section. Preexisting diabetes was significantly associated with caesarean section compared to GDM (OR 2.43). Five hundred fifty-one (40.1%) women underwent induction of labor, and 122 (22.1%) women had a secondary caesarean after IOL. Women induced due to spontaneous rupture of membrane (SROM) achieved the highest rate of VB at 93%. The lowest rates of VB occurred if indication for induction was for preeclampsia or hypertension. IOL was significantly less successful in preexisting diabetes with a VB achieved in 56.4% for type 1 diabetes and 52.6% of type 2 diabetes compared to GDM (78.2% in GDM; 81.2% in IGDM; OR 3.25, 95% CI 1.70–6.19, *p* < 0.001). The rate of VB was higher who were induced preterm compared to women with term IOL (*n* = 240 (81.9%) vs. *n* = 199 (73.2%); *p* < 0.05). Parity, previous VB and SROM favored VB after IOL, whereas preexisting diabetes, hypertension, and IOL after 40 + 0 weeks are independent risk factors for caesarean delivery.

## 1. Introduction

Diabetes affects an increasing number of pregnant women. According to recent studies, the prevalence for gestational diabetes mellitus (GDM) ranges between 5.4% and 11% in Europe [1, 2], whereas in America, over 6% are affected by GDM or some form of preexisting diabetes [3]. Women with preexisting diabetes (type 1 and type 2) and GDM are at higher risk of adverse maternal and neonatal outcome such as macrosomia and shoulder dystocia as well as hypertensive pregnancy disorders and primary caesarean section (CS). Women with type 1 or type 2 diabetes are at higher risk for congenital malformations and intrauterine death (IUD). The timing and mode of birth (MOB) in diabetic pregnancies depend primarily on maternal glucose control and fetal growth trajectory. However, there is no consensus on a uniform approach to the diagnosis and management of diabetes in pregnancy and timing and mode of delivery for all three forms of diabetes. Regarding diagnosis, the International Federation of Gynecology and Obstetrics, the Australasian Diabetes in Pregnancy Society (ADIPS), the Society of Obstetricians and Gynecologists of Canada (SOGC), and the American College of Obstetricians and Gynecologists (ACOG) recommend screening for all women between 24 and 28 weeks of gestation to diagnose gestational diabetes and subsequently start early and effective treatment, whereas the National Institute for Health and Care Excellence (NICE) only recommends screening for women with risk factors. There is also disagreement about earlier screening during pregnancy if there are risk factors and the interpretation of the 75 g oral glucose tolerance test [4]. The same disagreement prevails regarding time of birth. This lack of consensus is a major controversy in everyday obstetric practice [4]. Offering a routine induction of labor (IOL) at 37–39 weeks of gestation carries the potential benefit of reduced incidence of large for gestational age (LGA) babies and the associated complications such as shoulder dystocia and CS for failure to progress [5, 6]. On the other hand, routine IOL at 37–39 weeks of gestation might also lead to an increase risk of neonatal morbidity, fetal distress, or CS due to failed IOL [7]. The majority of recommendations focus on GDM, with less emphasis on outcomes for women with preexisting diabetes—therefore, birth outcomes for women with prepregnancy diabetes are lacking. Our primary aim was to compare the MOB in pregnant women with preexisting diabetes and GDM and possible factors influencing it. Secondarily, we aimed to evaluate obstetric and perinatal outcomes depending on MOB and type of diabetes (GDM vs. preexisting diabetes).

## 2. Material and Methods

We conducted a single center retrospective cohort study including all pregnant women with gestational and preexisting diabetes between 2015 and 2021 at a tertiary referral unit in Austria.

Twins and higher order multiples as well as patients with missing data or missing follow-up were excluded from the analysis.

All relevant data were acquired retrospectively using Viewpoint (perinatology database).

Routine pregnancy care at the department included first trimester screening scan between 11 and 14 weeks and anomaly scan between 19 and 24 weeks, which was performed by a fetal medicine specialist.

GDM was diagnosed with a standardized 75 g oral glucose tolerance test performed between 24- and 28-week gestation after a fast of at least 8 h, adhering to the criteria of The International Association of Diabetes and Pregnancy Study Groups (IADPSG) [8]. Women were instructed how to self-monitor their own blood glucose, and dietary recommendations were given to all women diagnosed with GDM during their first attendance at the diabetes clinic. Pharmacological treatment was initiated if lifestyle modification and dietary interventions failed, and women repeatedly recorded blood glucose levels above the glycemic target of fasting glucose *levels* > 95 mg/dl and/or 1-h postprandial glucose *levels* > 140 mg/dl. Pharmacological treatment included long-acting insulin, short-acting insulin, and/or metformin depending on the raised blood glucose levels. According to our local guidelines, we used metformin in some cases of GDM and preexisting diabetes, especially those with higher obesity who were insulin resistant. Details of metformin use can be found in the Table S1. All women were followed up regularly as per local guidelines until delivery. Women with preexisting diabetes and those with insulin-dependent GDM were monitored with fetal growth scan every 2 weeks and induced by 40-week gestation at the latest. Women with well controlled GDM without insulin therapy were followed up every 3 to 4 weeks and were induced depending on the estimated fetal weight and well-being of the woman at 40 weeks or later. If GDM was solely diet controlled with blood sugar levels in the normal range as well as no macrosomia was suspected, we did allow IOL after 40-week gestation with consent of the women. However, to minimize the risk of fetal death, they were followed up very closely after 40 weeks (every other day). MOB was categorized into vaginal birth (VB), assisted birth (vacuum extraction only as forceps are not utilized in our department), secondary CS (CS after a trial of IOL or during labor), and elective CS. VB after CS was only offered and discussed with women after assessing known factors affecting successful VB such as estimated fetal *weight* < 90*th* centile, maternal age, previous VB, body mass index (BMI), and indication of the previous CS. The primary outcome was successful VB. Secondary outcomes included rates of IOL, rates of CS, birthweight, shoulder dystocia IUD, perinatal death, and fetal structural malformation.

Ethical approval was obtained from the Ethics Committee of the Medical University of Vienna (EK1368/2022). This manuscript was structured according to the STROBE guidelines (adapted for observational studies using routinely collected health data).

### 2.1. Data Analysis

For statistical analysis, metric normally distributed variables were presented as the *mean* ± *SD* or median and IQR. Metric variables were compared using Student's *t*-test in the case of a standard distribution and Mann–Whitney *U*-test in the case of variables not following a normal distribution [9]. We used a chi-square test to compare binary and categorical variables. A two-sided *p* value of < 0.05 was set for statistical significance. Binary logistic regression was used to assess factors associated with vaginal delivery. A multivariate logistic regression model was stepwise fitted to test factors associated with a successful IOL. Covariates included gestational age (GA) at induction, previous vaginal delivery, preexisting diabetes mellitus, BMI, age, and preeclampsia. For this analysis, the variable GA at induction was divided into two groups before and after 40 weeks. Statistical analyses were performed using IBM SPSS Statistics version 23 [9].

## 3. Results

### 3.1. Baseline Patient Characteristics

Overall, 1385 singleton pregnancies complicated by diabetes (preexisting and gestational diabetes) were evaluated. The average age of affected women was 33 years, ranging from 14 to 52 years, with an average BMI before pregnancy of 27.6 kg/m^2^ and an average weight gain of 7.27 kg (SD of ± 4.33). Patients' characteristics are summarized in Table 1.

Mean GA at birth was 38.8 weeks (range 23 + 2–42 + 5). Of 1385 singleton pregnancies, 1373 were liveborn. There were 11 intrauterine fetal deaths (IUFDs), and one woman opted for termination of pregnancy because of a fetal structural malformation (abdominal wall defect with bladder extrophy). There were three early neonatal deaths within 7 days of birth, including one preterm delivery at 23 weeks weighing 575 g, one fetus with tracheal agenesis, and one case with uncomplicated delivery at 37 weeks developing bacterial sepsis after birth. In 94 (6.8%) cases, a structural malformation was detected prenatally in the fetus. The most common structural malformations were congenital heart defects (*n* = 41, 43.6%) followed by anomalies of the urogenital system (*n* = 13, 13.8%) cases. Neonatal characteristics and outcomes are summarized in Table 2.

### 3.2. MOB

Overall, 1022 (74.4%) women had VB, including 96 (7%) with an assisted birth (ventouse); 351 (25.6%) of women were delivered by CS including 27 (2.0%) as an emergency CS category 1 CS. Rates of VB versus elective and secondary caesarean varied widely depending on the type of diabetes (Table 3). Overall, preexisting diabetes was significantly associated with CS compared to gestational diabetes (*p* < 0.01).

Of 101 women that had one previous CS, 63 women opted for VB after CS. Subsequently, 49 (77.8%) women had a successful vaginal delivery.

### 3.3. IOL

Five hundred fifty-one (40.1%) women had IOL between 31.7- and 41.4-week GA, mean 39.4 weeks (*SD* ± 1.16). The main indication for IOL was the diagnosis of insulin-dependent gestational diabetes (*n* = 184, 33.4%). Findings are summarized in Table 4.

Four hundred twenty-nine of women in the IOL group (77.9%) out of 551 women had a VB, whereas 122 (22.1%) women had a secondary caesarean after commencing IOL. IOL due to SROM was the most successful with respect to the primary outcome with 93.0% of women achieving a VB. The lowest rate of VB occurred in women who were diagnosed with preeclampsia or hypertension during pregnancy and underwent induction as a result.

Importantly, IOL was significantly less successful in preexisting diabetes (vaginal delivery: DM1 56.4%; DM2 52.6%) compared to gestational diabetes (78.2% in GDM; 81.2% in IGDM).

The rate of successful VB was higher in women that had IOL before 40 weeks (34 to 39 + 6 weeks) compared to women induced after 40-week gestation—IOL at 40 + 0 to 41 + 3 weeks (240 (81.9%) vs. 199 (73.2%); *p* < 0.05), respectively.

Parity and SROM were significantly associated with successful VB, whereas the diagnosis of preeclampsia was significantly associated with a birth by CS. Encouragingly, neither age nor BMI of the pregnant women or weight gain during pregnancy and fetal malformation were significantly associated with having a CS after IOL.

Multivariate statistic logistic regression was used to identify risk factors for a birth by CS after IOL. While previous VB unsurprisingly favored subsequent VB after IOL in the index pregnancy, preexisting diabetes mellitus, IOL after 40-week gestation and preeclampsia were all independent risk factors for secondary CS (Table 5).

There were two women with shoulder dystocia after IOL, one with DM1 with a birth weight of 4345 g (95th centile) and one woman with IGDM with a birth weight of 4040 g (81st centile).

The main indication for CS after IOL was a pathological intrapartum CTG trace in 63 (51.6%) cases, followed by inefficient IOL in 29 cases (23.8%).

### 3.4. CS

Three hundred fifty-one (25.6%) offspring were delivered by CS. One hundred twenty-eight cases (36.5%) had an elective CS due to previous CS in 61 (47%) women (23 women had at least two previous CS), fetal structural malformation in 20 (15.9%) pregnant women, and malpresentation (breech, transverse) in 19 (15.1%) pregnant women. One hundred ninety-six of 351 women having a CS attempt to give birth vaginally. The main indications were a pathological CTG trace during labor in 116 pregnant women (51.6%) or failed to progress in 53 (23.6%) pregnant women. Women with preexisting diabetes (DM1 28.3%, DM2 25.0%) had a significantly higher rate of secondary CS compared to women with gestational diabetes (GDM 13.3%, IGDM 13.5%). There were no significant differences in the rate of emergency CS between preexisting and gestational diabetes. Additionally, we could not observe an association between metformin therapy and CS.

## 4. Discussion

Our aim was to compare time and MOB in women pregnant with a singleton fetus where the pregnancy was complicated by the diagnosis of GDM or where they had preexisting diabetes. In total, 1022 (74.4%) women achieved a VB, and 351 (25.6%) gave birth by CS. We report an overall rate of IOL in this high-risk cohort of 40.1% (*n* = 551) of whom 429 had a successful VB. Women with preexisting diabetes had significantly higher rate of CS after IOL compared to women with GDM. Although in our cohort the main indication for CS is previous CS in all four groups, women with preexisting diabetes had significantly more often an unplanned CS compared to women with gestational diabetes. With the recommendation for women with type 1 and type 2 diabetes to give birth between 38 and 40 weeks of gestation to avoid intrauterine IUD and reduce the incidence of macrosomia, this inevitably increases the number of CS [10, 11]. We observed the highest rate of CS in women with preexisting DM (type 1 and type 2 diabetes with 45.3% and 41.7%, respectively); these rates are lower compared to previously published evidence in a similar patient cohort with 52% to 55% [10, 12]. This might be explained by the low rates of elective CS in our cohort and the fact that the majority of women with preexisting diabetes (68.5%) had at least one previous VB.

Fischer et al. and Kjos, Berkowitz, and Xiang demonstrated that previous CS and nulliparity and hypertensive disorders were independent predictors for emergency CS in pregnant women with preexisting diabetes [13, 14]. Another recent retrospective analysis from 2022 by Weschenfelder et al. described that a previous VB and reduced weight gain during pregnancy were independent predictors for a successful VB in women with type 1 diabetes, whereas the time since diagnosis of diabetes was negatively associated with successful VB [15]. We also observed in our cohort that IOL after 40 weeks, preeclampsia, and preexisting diabetes were independent risk factors for CS whereas neither maternal age nor BMI was significantly associated with having a CS: This evidence contradicts previous published data and therefore is important to consider when counselling women with respect to the likelihood of a successful outcome when offering them an IOL. Regarding the specific association between age, BMI, and insulin resistance during pregnancy, a recent work by Mirabelli et al. showed that the risk of developing GDM is more impacted by preconception BMI rather than age [16] and should be included in counselling women with high risk for GDM or history of previous GDM. Nevertheless, this study provides evidence that maternal preconception BMI is an important fact in determining GDM but does not address the aspect of IOL depending on BMI and/or age. IOL prior to 40 weeks may be controversial; however, we demonstrate a higher success rate for achieving a vaginal delivery in women induced prior to 40 weeks. Generally, IOL prior to 39-week gestation will reduce the incidence of CS after IOL and of macrosomia and its attendant risk in a diabetic cohort of shoulder dystocia [5, 6, 17].

However, IOL at 37–39 weeks of gestation might also lead to an increase risk of neonatal morbidity, fetal distress, or CS due to failed IOL [7]. Melamed et al. reported that IOL before < 39-week gestation led to a lower CS rate but was associated with a rise in admission to neonatal intensive care unit [17].

In contrast, another work could prove that there is a similar risk for CS regardless of IOL or expectant management between 37 and 40 weeks of gestation, but a 3-fold rise in the number of CS rate at 40 weeks and beyond [18, 19]. Rates of CS differed based on cervical exam and parity. Risk of CS was higher with a Bishop score < 5 and in nulliparous women. The authors conclude from this that GA alone does not significantly impact maternal and neonatal outcomes [18]. In our study, the main reason for IOL was the diagnosis of insulin-dependent gestational diabetes (33.4%); women induced due to SROM had the highest rate of VB at 93%. The lowest rates of VB after induction occurred if the indication for IOL was preeclampsia or hypertension. This is in line with the current published literature's findings that hypertensive disorders are associated with emergency CS, regardless of type of diabetes or glycemic control [13].

A recent randomized control trial by published in 2017 demonstrated that in addition to SROM, maternal age, parity, advanced GA at IOL, and Bishop score are among the predictive factors that favor VB after induction [20]. It is important to consider that with rising numbers of women with preexisting diabetes becoming pregnant and higher rates of women developing gestational diabetes, VB is preferable in this cohort due to its lower complication risk. Furthermore, it is also noteworthy the findings regarding metformin use in pregnancy and possible claimed association of increased number of CS and preterm birth before 37 weeks of gestation. However, we could not find a significant association which would lead to this assumption. Other studies confirmed our results: The studies by Tocci et al., Tarry-Adkins, Ozanne, and Aiken and Rowan et al. found no significant difference in the rate of preterm birth (birth before 37 weeks) between women with GDM who received metformin therapy and those who received insulin therapy [21–23]. Instead, metformin seems to offer some benefits such as preventing the development of gestational hypertension, preeclampsia, and subsequently risk of preterm delivery and transfer to the NICU [21]. On the other hand, these data indicate that metformin can negatively affect the fetal body composition and intrauterine growth [21, 22]. However, regarding the work by Rowan et al., metformin (alone or with supplemental insulin) is not associated with increased perinatal complications as compared with insulin [23]. Nevertheless, as yet, we do not have a validated CS prediction model in women with diabetes in pregnancy; therefore, the findings of our paper will aid in the counselling of women wishing to embark on an IOL. Further, there is still no consensus between expected management and IOL for pregestational and gestational diabetes, and the existing data is still very scarce, and further prospective studies, as well as RCT studies, are required to optimize time and MOB in diabetic pregnancies.

The main strengths of this work include the large sample size at a single center providing standardized care using international guidelines for diagnosis of GDM, which allow for the results of this cohort to be extrapolated to other international centers. Additionally, we could demonstrate the benefit of IOL before 40 weeks which is helpful when counselling women. However, the retrospective design of the study can be seen as main limitation.

## 5. Conclusions

In conclusion, this work illustrates factors that influence the MOB after IOL in diabetic women both positively and negatively. Factors such as parity, previous vaginal delivery, and SROM favor vaginal delivery after IOL, whereas preexisting diabetes, hypertensive pregnancy disorders, and IOL after 40 + 0 weeks of gestation are independent risk factors for CS. Those factors should be considered when planning time and MOB in diabetic pregnant women.

## Figures and Tables

**Table 1:** Patients' characteristics.

	**n** **(%)**	*Mean* ± *SD*	**Median**	**CGM use** **n** **(%)**	**Average weight gain (kg)**	**Glycemic targets achieved** **n** **(%)**
Maternal age (years)		33 ± 6				
BMI		27.6 ± 6.2				
< 25	521 (37.6%)					
25–29.9	411 (33.3%)					
> 30	403 (29.1%)					
Gravida			3			
Para			2			
Smoking						
Yes	164 (11.8%)					
No	1221 (88.2%)					
Conception						
Spontaneous	1319 (95%)					
ART	70 (5%)					
Different forms of diabetes						
Diabetes mellitus type 1	53 (3.9%)			42 (79.2%)	6.12	48 (90.6%)
Diabetes mellitus type 2	36 (2.6%)			14 (38.9%)	8.36	31 (86.1%)
Gestational diabetes (diet)	759 (55.3%)			0 (0%)	7.17	720 (94.9%)
Gestational diabetes (insulin dependent)	525 (38.2%)			25 (4.8%)	7.32	473 (90.1%)

**Table 2:** Neonatal characteristics.

	**n** **(%)**	**Min**	**Max**	**M** **e** **a** **n** ± **SD**
Gestational age at delivery (weeks)		23.3	427	38.9 ± 1.9
Preterm delivery				
Yes	143 (10.4%)			
No	1230 (89.6%)			
Birth weight (g)		575	4960	3335.8 ± 579.8
LGA (*weight* > 90*th* centile)				
Diabetes mellitus type 1	13 (24.5%)			
Diabetes mellitus type 2	12 (33.3%)			
Gestational diabetes (diet)	42 (5.6%)			
Gestational diabetes (insulin dependent)	76 (14.5%)			
SGA (*weight* < 10*th* centile)				
Diabetes mellitus type 1	3 (5.7%)			
Diabetes mellitus type 2	2 (5.6%)			
Gestational diabetes (diet)	84 (11.1%)			
Gestational diabetes (insulin dependent)	51 (9.7%)			
Birth length (cm)		31	60	51 ± 3.2
NICU admission				
Yes	142 (10.3%)			
No	1231 (89.7%)			

**Table 3:** Mode of delivery in patients affected by different types of diabetes.

**Type of diabetes**	**Vaginal delivery**	**Assisted birth**	**Planned CS**	**Secondary CS**	**Emergency CS**	**Shoulder dystocia**
DM type 1	25 (47.2%)	3 (5.7%)	8 (15.1%)	15 (28.3%)	1 (1.9%)	1 (1.9%)
DM type 2	19 (52.8%)	0	4 (11%)	9 (25.0%)	2 (5.6%)	2 (5.6%)
Gestational diabetes	515 (67.9%)	61 (8.0%)	69 (9.1%)	101 (13.3%)	13 (1.7%)	0
Insulin-dependent gestational diabetes	363 (69.1%)	32 (6.1%)	47 (9.0%)	71 (13.5%)	11 (2.1%)	1 (0.2%)
Overall	922 (67%)	96 (7.0%)	128 (9.3%)	196 (14.3%)	27 (2.0%)	4 (0.3%)

**Table 4:** Indication for induction of labor.

**Indication for induction**	**N** **(%)**	**Median gestational age at IOL**	**Vaginal delivery**
IGDM	184 (33.4%)	40.00	143 (77.7%)
GDM	139 (25.2%)	40.00	116 (83.5%)
Macrosomia (*AC* > 95*th* centile or *EFW* > 95*th* centile)	74 (13.4%)	39.00	53 (71.6%)
SROM	43 (7.8%)	38.43	40 (93.0%)
Hypertension/preeclampsia	93 (7.1%)	38.29	22 (56.4%)
IUGR, path Doppler	17 (3.1%)	38.71	13 (76.5%)
Insufficient DM treatment	16 (2.9%)	38.86	14 (87.5%)
Previous IUFD	11 (2.0%)	37.71	8 (72.7%)
DM1	11 (2.0%)	39.00	6 (54.5%)
Maternal disease	7 (1.3%)	38.29	7 (100%)
Prolonged pregnancy	7 (1.3%)	41.43	5 (71.4%)
DM2	3 (0.5%)	39.14	2 (66.7%)

**Table 5:** Multivariate logistic regression analysis predicting risk factors for CS after IOL.

**Multivariate analysis**	**OR**	**95% CI**	**p** **value**
Preexisting DM	4.76	2.30–9.84	*p* < 0.001
GA at induction (> 40 weeks)	2.27	1.39–3.73	*p* < 0.001
Preeclampsia	4.35	1.51–12.57	*p* < 0.01
Parity	0.74	0.62–0.87	*p* < 0.001

## Data Availability

The data presented in this study are available on request from the corresponding author. The data are not publicly available due to data privacy.
