# Supplementary material for: Factors Affecting Mode of Birth in Women With Preexisting Diabetes and Gestational Diabetes: A Retrospective Cohort at a Tertiary Referral Center
Source: J Diabetes Res. 2024 May 31;2024:5561761. doi: 10.1155/2024/5561761 (PMC11178421; doi:10.1155/2024/5561761)
Supplement: Supporting Information — Additional supporting information can be found online in the Supporting Information section. Table S1 Metformin use in patients affected by different types of diabetes. [file 5561761.f1.docx]

**S1. Met**formin use in patients affected by different types of diabetes

| Type of diabetes (n) | Metformin therapy N (%) | Average Metformin dosage(mg)/d (SD) |
| --- | --- | --- |
| DM type 1 | 2 (3,8%) | 1000 |
| DM type 2 | 20 (55,6%) | 1491,7 (+/-671,8) |
| Gestational diabetes | 73 (9,6%) | 1461,2 (+/-508,0) |
| Insulin dependent gestational diabetes | 52(9,9%) | 1747,6(+/-563,2) |
